# Identification and expression analysis of genes related to calyx persistence in Korla fragrant pear

**DOI:** 10.1186/s12864-016-2470-3

**Published:** 2016-02-24

**Authors:** Maosong Pei, Jianxin Niu, Chenjing Li, Fujun Cao, Shaowen Quan

**Affiliations:** Department of Horticulture, College of Agriculture, Shihezi University, Shihezi, 832003 Xinjiang China; Xinjiang Production and Construction Corps Key Laboratory of Special Fruits and Vegetables Cultivation Physiology and Germplasm Resources Utilization, Shihezi, 832003 Xinjiang China

**Keywords:** Persistent calyx, Deciduous calyx, Transcriptome sequencing, DGE sequencing

## Abstract

**Background:**

The objective of this study was to increase understanding about genetic mechanisms affecting calyx persistence in Korla fragrant pear (*Pyrus brestschneideri* Rehd). Flowers were collected at early bloom, full bloom, and late bloom. The RNA was extracted from the flowers and then combined according to calyx type. Transcriptome and digital gene expression (DGE) profiles of flowers, ovaries, and sepals with persistent calyx (SC_hua, SC_ep, and SC_zf, respectively) were compared with those of flowers, ovaries, and sepals with deciduous calyx (TL_hua, TL_ep, and TL_zf, respectively). Temporal changes in the expression of selected genes in floral organs with either persistent or deciduous calyx were compared using real-time quantitative PCR (qRT-PCR).

**Results:**

Comparison of the transcriptome sequences for SC_hua and TL_hua indicated 26 differentially expressed genes (DEGs) with known relationship to abscission and 10 DEGs with unknown function. We identified 98 MYB and 21 SPL genes from the assembled unigenes. From SC_zf vs TL_zf, we identified 21 DEGs with known relationship to abscission and 18 DEGs with unknown function. From SC_ep vs TL_ep, 12 DEGs with known relationship to abscission were identified along with 11 DEGs with unknown function. Ten DEGs were identified by both transcriptome sequencing and DGE sequencing.

**Conclusions:**

More than 50 DEGs were observed that were related to calyx persistence in Korla fragrant pear. Some of the genes were related to cell wall degradation, plant hormone signal transduction, and stress response. Other DEGs were identified as zinc finger protein genes and lipid transfer protein genes. Further analysis showed that calyx persistence in Korla fragment pear was a metabolic process regulated by many genes related to cell wall degradation and plant hormones.

**Electronic supplementary material:**

The online version of this article (doi:10.1186/s12864-016-2470-3) contains supplementary material, which is available to authorized users.

## Background

Korla fragrant pear is one of the most valuable fruits in China’s Xinjiang Province [1]. The calyx of Korla fragrant pear is sometimes persistent. This can negatively affect pear shape and quality. Previous studies about Korla fragrant pear have examined the relationship between calyx persistence and cultivation practice [2], tree vigor [2], pollen source [3–5], growth regulators [6–8], and plant nutrition [9]. Some studies have investigated the molecular mechanisms for calyx persistence in Korla fragrant pear. For example, Dong et al. and Wang et al. cloned a kfpMYB gene related to calyx persistence using differential display RT-PCR [10, 11]. Qi et al. used digital transcript abundance measurements to identify genes correlated with calyx abscission [12].

High-throughput sequencing has contributed greatly to the study of gene function in non-model plants. High-throughput sequencing makes it possible to understand the genome and the transcriptome of a species more comprehensively [13–15]. High-throughput sequencing of RNA (RNA-Seq) has been successfully applied in *Malus domestica* [16, 17], *Myrica rubra* [18, 19], *Vaccinium section Cyanococcus* [20], *Litchi chinensis* Sonn [21], *Pyrus bretschneideri* Rehd [22], *Vitis vinifera cv. Shiraz* [23], *Musa acuminate* [24, 25], *Citrus sinensis* [26, 27], *Prunus persica* [28], and *Diospyros kaki* [29]. The objective of this experiment was to identify candidate genes related to calyx persistence in Korla fragrant pear using both transcriptome and digital gene expression (DGE) sequencing.

## Results and discussion

### Transcriptome sequencing and assembly

**Fig. 1:**
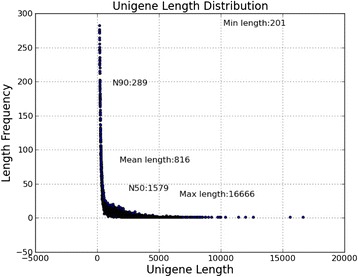
Length distribution of the assembled unigenes

In total, 107202492 raw reads were generated by Illumina sequencing of SC_hua vs TL_hua (Table 1). There were 103466288 clean reads after removing low-quality sequences. Assembly of the clean reads resulted in 39891341 unigenes ranging in size between 201 and 16666 bp (Fig. 1). The N50 length of the unigenes was 1579 bp and the N90 length was 289 bp.

**Table 1:** Summary of the sequence analyses

Sample	Raw Reads	Clean Reads	Clean Bases	Error (%)	Q20 (%)	Q30 (%)	GC Content (%)
SC_hua_1	27216916	26238309	2.62G	0.03	98.51	94.71	47.18
SC_hua_2	27216916	26238309	2.62G	0.04	96.71	91.53	47.24
TL_hua_1	26384330	25494835	2.55G	0.03	98.55	94.82	46.87
TL_hua_2	26384330	25494835	2.55G	0.04	96.79	91.67	46.93
Summary	107202492	103466288	10.34G				

Sample: Sample name_1, left reads; Sample name_2, right reads. The total number of clean reads is left + right. Clean reads: The number of reads after removing low-quality sequences. The subsequent analysis is based on clean reads. Error rate: Base error rate.Q20 and Q30, the percentage of bases with Phred values >20 and >30, respectively. GC content: the GC ratio of the total base number

### Sequence annotation

The unigenes were aligned with seven public databases [i.e., NR (NCBI non-redundant protein sequences), NT (NCBI nucleotide sequences), KEGG (Kyoto Encyclopedia of Genes and Genomes), SwissProt (A manually annotated and reviewed protein sequence database), PFAM (Protein family), GO (Gene Ontology) and KOG/COG (Clusters of Orthologous Groups of proteins)] (Table 2). The results showed that 18605 unigenes (38.05 %) had significant matches in the NR database, 16700 unigenes (34.15 %) had significant matches in the NT database, and 17326 unigenes (35.43 %) had significant matches in the SwissProt database. In total, 26088 unigenes (53.35 %) were annotated in at least one database, with 3037 unigenes (6.21 %) being annotated in all seven databases.

**Table 2:** The success rate of gene annotation

	Number of Unigenes	Percentage (%)
Annotated in NR	18605	38.05
Annotated in NT	16700	34.15
Annotated in KO	6925	14.16
Annotated in SwissProt	17326	35.43
Annotated in PFAM	16935	34.63
Annotated in GO	17749	36.3
Annotated in KOG	8891	18.18
Annotated in all Databases	3037	6.21
Annotated in at least one Database	26088	53.35
Total Unigenes	48894	100

Annotated in NR: The unigene number and annotation rate in the NR database. Annotated in NT: The unigene number and annotation rate in the NT database. Annotated in KO: The unigene number and annotation rate in the KO database. Annotated in SwissProt: The unigene number and annotation rate in the SwissPort database. Annotated in PFAM: The unigene number and annotation rate in the PFAM database. Annotated in GO: The unigene number and annotation rate in the GO database. Annotated in KOG: The unigene number and annotation rate in the KOG database. Annotated in all Databases: The unigene number and annotation rate in all seven databases. Annotated in at least one database: The unigene number and annotation rate in at least one database

**Fig. 2:**
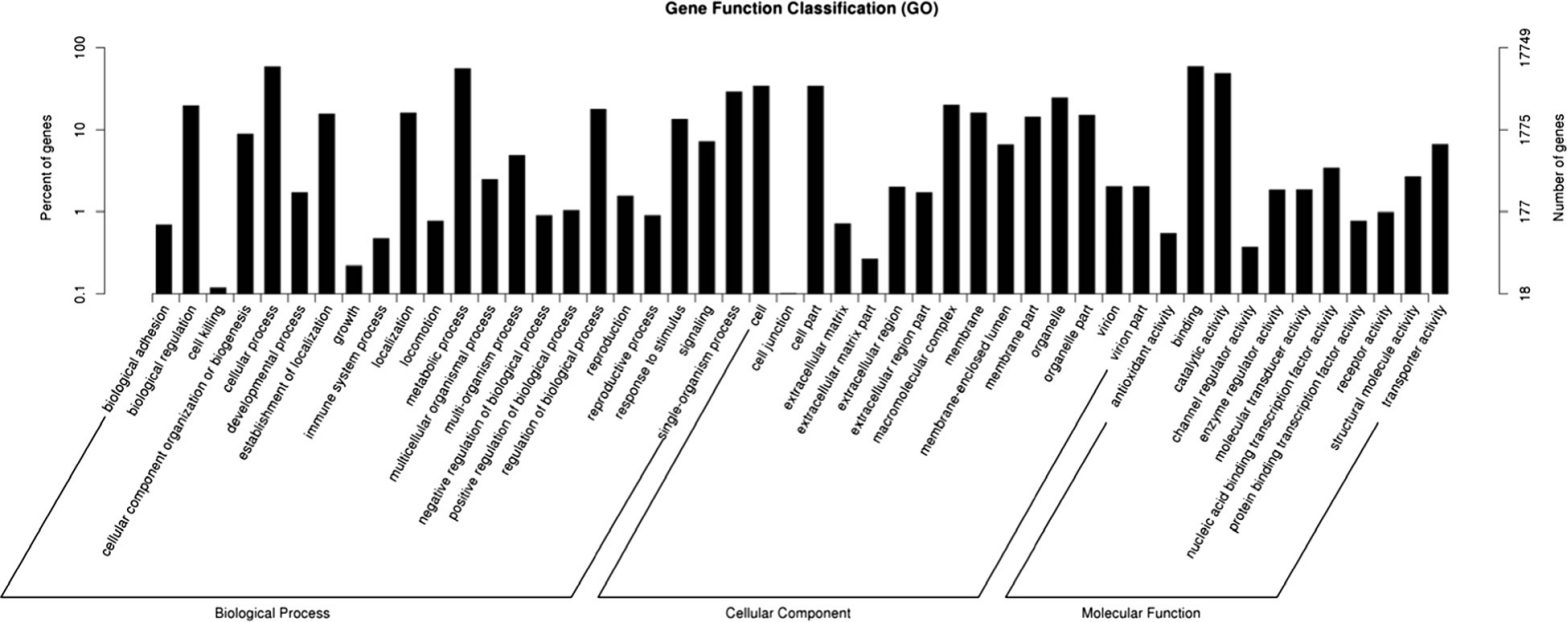
GO categorization of unigenes

A total of 17749 unigenes were subjected to GO analysis (Fig. 2). In the cellular component (CC) category, genes involved in ‘cell’ (6093), ‘cell part’ (6087), and ‘organelle’ (4357), were highly represented. The molecular function category (MF) mainly included genes involved in ‘binding’ (10493), ‘catalytic activity’ (8571) and ‘transporter activity’ (1176). In the biological process (BP) category, ‘cellular process’ (10437), ‘metabolic process’ (9848) and ‘single-organism process’ (5155) were highly represented.

**Fig. 3:**
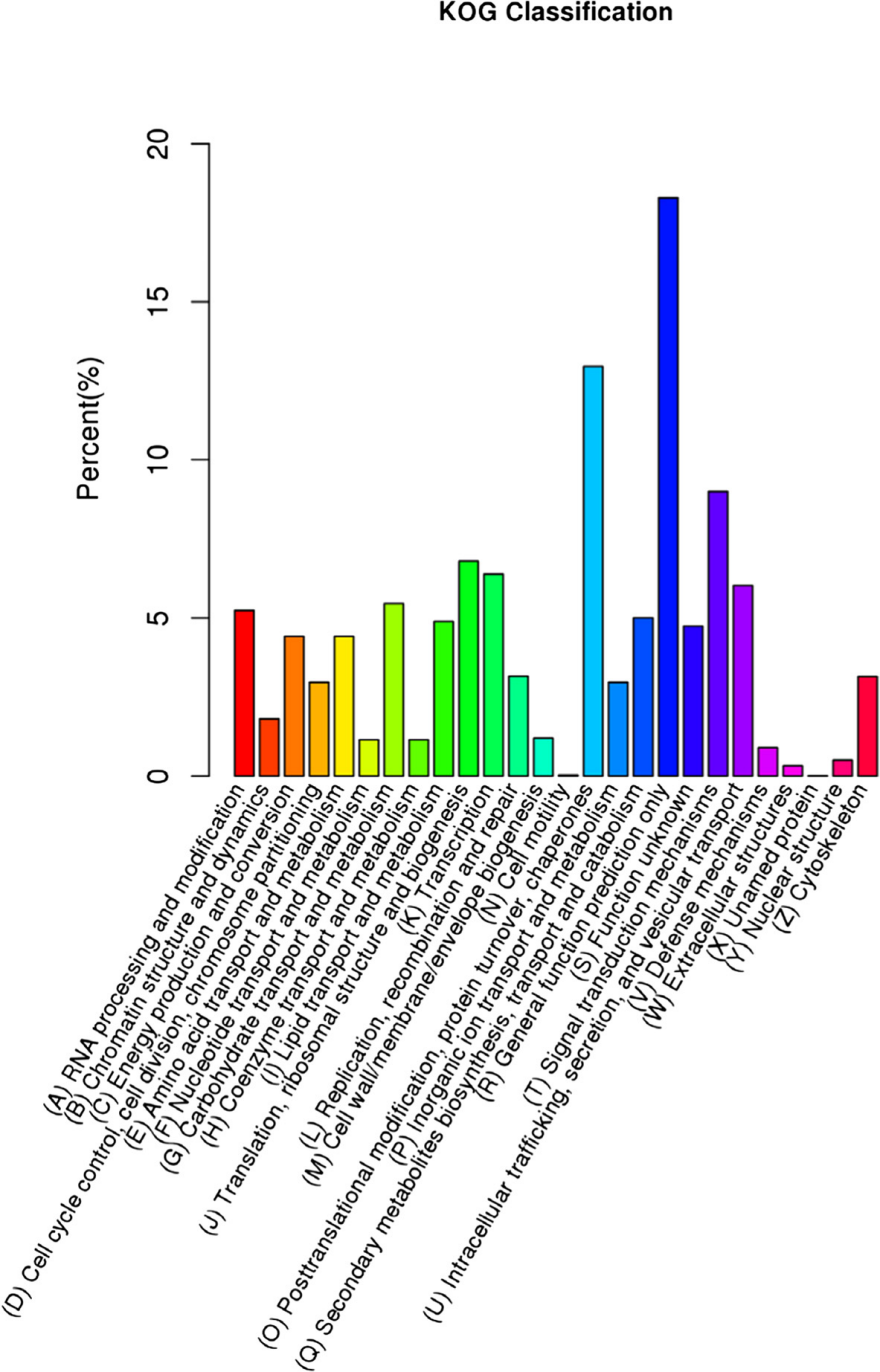
KOG annotation of putative proteins

The unigenes were all subjected to a search against the COG database for functional prediction and classification. In total, 8891 unigenes were assigned to COG classification and divided into 26 specific categories (Fig. 3). The largest group was ‘general function prediction only’ (1626), followed by ‘post-translational modification, protein turnover, chaperones’ (1152), ‘signal transduction mechanisms’ (800), ‘intracellular trafficking, secretion, and vesicular transport’ (535), and ‘carbohydrate transport and metabolism’ (485). Only a few unigenes were assigned to ‘extracellular structures’ (28) and ‘cell motility’ (3).

**Fig. 4:**
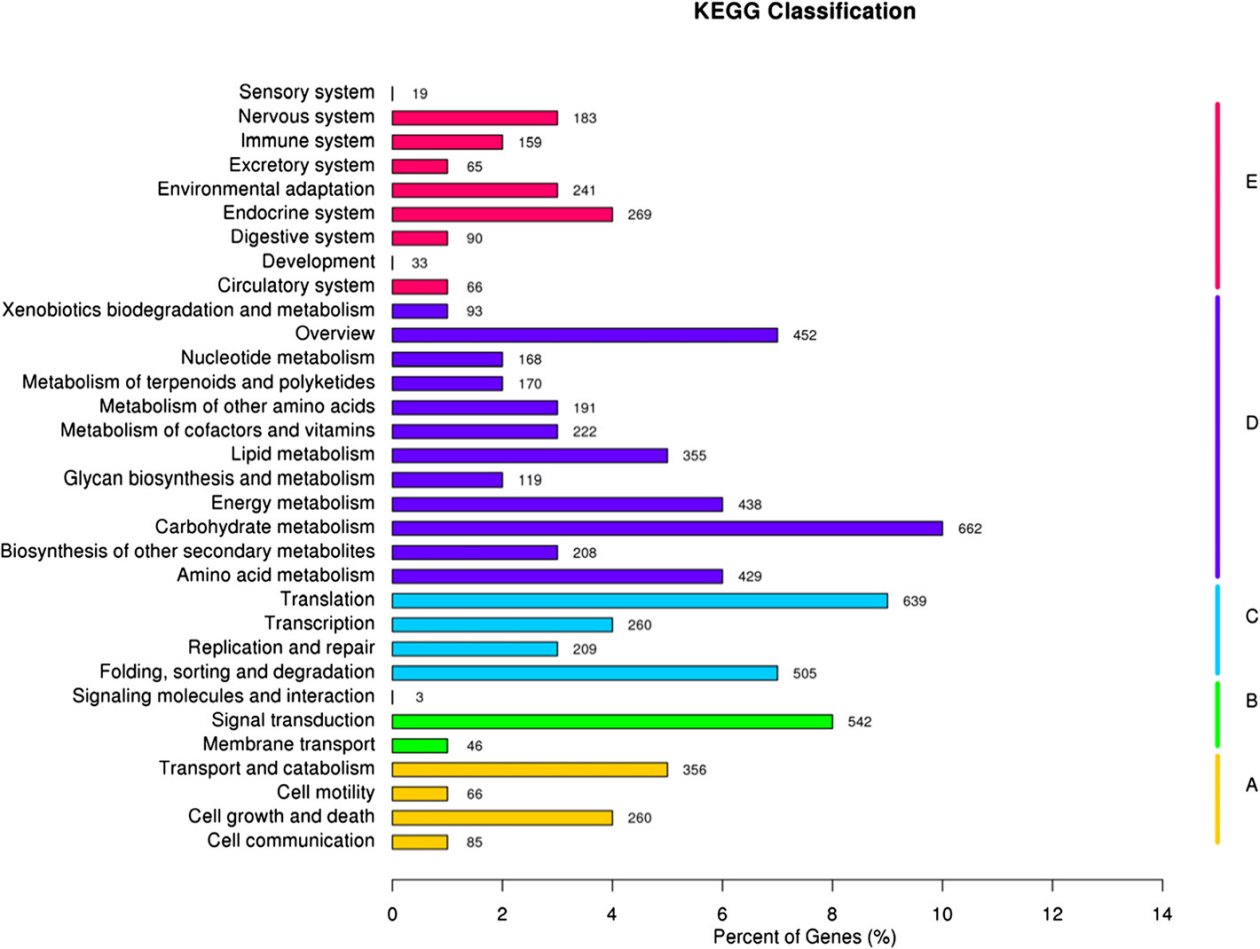
KEGG annotation of putative proteins

Unigene metabolic pathway analysis was also conducted using KEGG. This process predicted a total of 258 pathways, representing 6925 unigenes (Fig. 4). The pathways involving the highest number of unique transcripts were ‘carbohydrate metabolism’ (662), followed by ‘translation’ (639) and ‘signal transduction’ (542). The above data is a very valuable genetic resource for studying calyx persistence in Korla Fragrant Pear.

### Differential expression analysis in SC_hua vs TL_hua

**Fig. 5:**
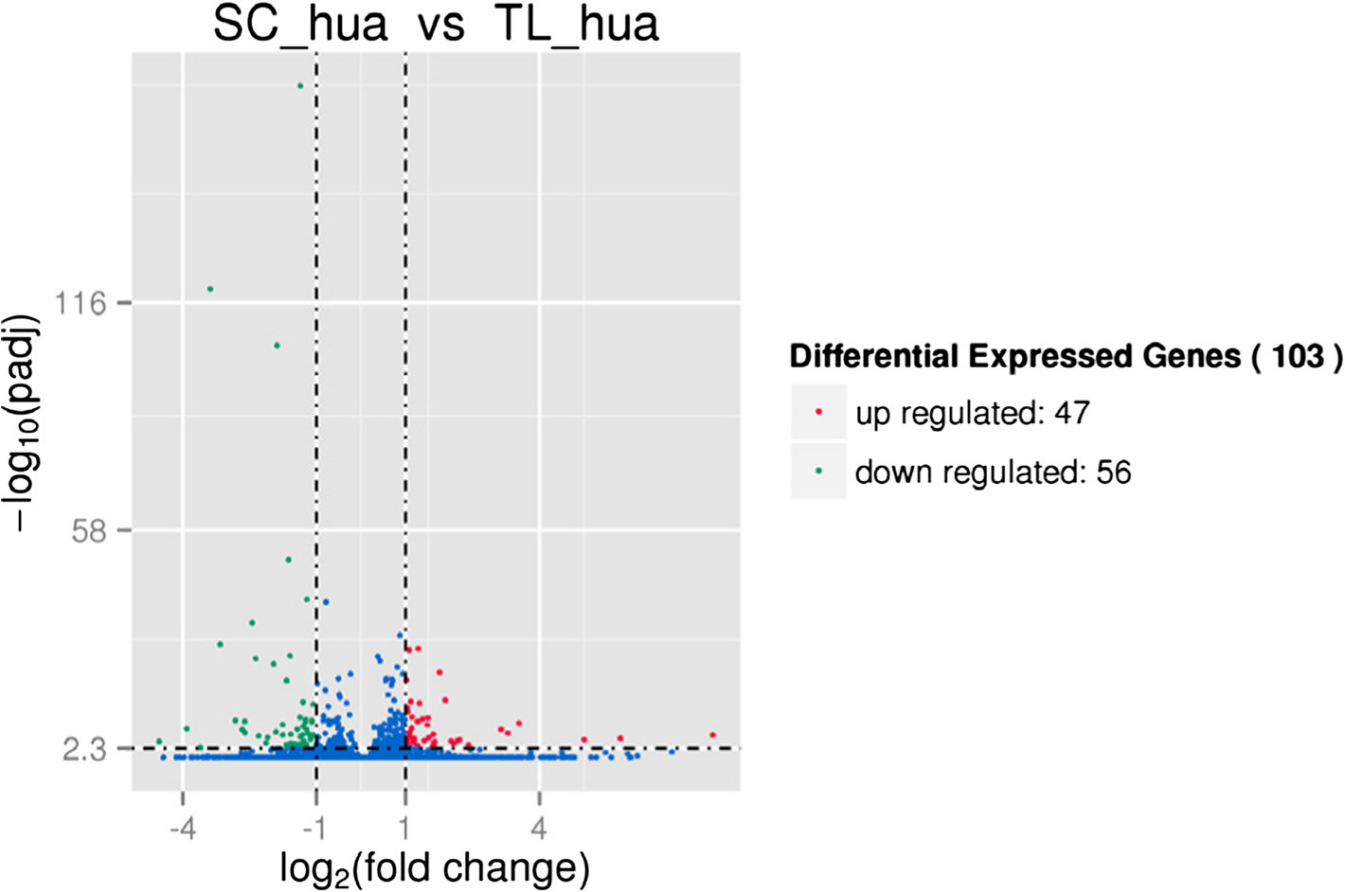
Up-regulated and down-regulated differentially expressed genes in SC_hua vs TL_hua

Differentially expressed genes (DEGs) are defined as genes that are significantly enriched or depleted in one sample relative to another (q value < 0.005 and |log2 (foldchange)| >1). In the rest of this paper, up-regulated means that the gene expression level was greater in samples with persistent calyx than in samples with deciduous calyx. Down-regulated means that the gene expression level was less in samples with persistent calyx than in samples with deciduous calyx. There were 103 DEGs among 48894 unigenes in SC_hua vs TL_hua. Among these, 47 DEGs were up-regulated and 56 DEGs were down-regulated (Fig. 5).

To further characterize the function of the DEGs, GO enrichment analysis was conducted for all of the DEGs in SC_hua vs TL_hua with the whole transcriptome as the background (Additional file 1). In the BP category, the top three enriched terms were ‘heterocycle biosynthetic process’, ‘organic cyclic compound biosynthetic process’ and ‘cellular nitrogen compound biosynthetic process’. In the CC category, ‘nuclear part’, ‘membrane-enclosed lumen’, ‘intracellular organelle lumen’, ‘organelle lumen’ and ‘nuclear lumen’ were the dominant enriched terms. In the MF category, ‘nucleic acid binding transcription factor activity’ and ‘sequence-specific DNA binding transcription factor activity’ were most highly enriched. A GO enrichment analysis was also conducted for the up-regulated DEGs (Additional file 2). In the BP category, ‘biological regulation’, ‘regulation of biological process’, and ‘regulation of cellular process’ were most highly enriched. In the CC category, ‘membrane-enclosed lumen’, ‘intracellular organelle lumen’, ‘organelle lumen’ and ‘nuclear lumen’ were the main enriched terms. In MF, the top two enriched terms were ‘nucleic acid binding transcription factor activity’ and ‘sequence-specific DNA binding transcription factor activity’.

The KEGG pathway enrichment analysis for DEGs also revealed both common and tissue specific patterns of over-representation (Additional file 3). The top-four enriched pathways for DEGs in SC_hua vs TL_hua were ‘cysteine and methionine metabolism’, ‘porphyrin and chlorophyll metabolism’, ‘phenylalanine metabolism’ and ‘isoquinoline alkaloid biosynthesis’. For up-regulated DEGs (Additional file 4), ‘calcium signaling pathway’, ‘porphyrin and chlorophyll metabolism’, ‘phosphatidylinositol signaling system’ and ‘glycerolipid metabolism’ were most highly enriched. For down-regulated DEGs (Additional file 5), ‘cysteine and methionine metabolism’, ‘isoquinoline alkaloid biosynthesis’ and ‘biosynthesis of amino acids’ were the three main enriched pathways.

### DGE sequencing

A DGE analysis was performed to compare gene expression in SC_ep, SC_zf, TL_ep, and TL_zf. After removing low-quality sequences, we obtained 12283115, 10084701, 9449491 and 9999449 clean reads in SC_ep, SC_zf, TL_ep, and TL_zf, respectively (Table 3). The clean data were mapped back onto the assembled transcriptome using RSEM software. The bowtie parameter mismatch was 2. Among the four DGE sequencing results, at least 91.50 % of the sequences could be mapped back to the reference sequences (Table 4).

**Table 3:** Statistics of DGE sequencing

Sample	Raw Reads	Clean Reads	Clean Bases	Error (%)	Q20 (%)	Q30 (%)	GC Content (%)
SC_ep	12343471	12283115	0.61G	0.01	99.21	97.67	46.89
SC_zf	10138431	10084701	0.5G	0.01	99.2	97.62	46.98
TL_ep	9486992	9449491	0.47G	0.01	99.24	97.74	46.69
TL_zf	10139423	9999449	0.5G	0.01	99.22	97.7	46.79

Clean reads: The number of reads after removing low-quality sequences. The subsequent analysis is based on clean reads. Error rate: Base error rate. Q20 and Q30, the percentage of bases with Phred values >20 and >30, respectively. GC content: the GC ratio of the total base number

**Table 4:** DGE reads mapped to the reference sequences

Sample name	Total reads	Total mapped
SC_ep	12283115	11280554 (91.84 %)
SC_zf	10084701	9248894 (91.71 %)
TL_ep	9449491	8646697 (91.50 %)
TL_zf	9999449	9172179 (91.73 %)

Total reads: Number of reads after removing low-quality sequences (clean data). Total mapped: Number of reads that could be mapped back to the reference sequences. Values within the parenthesis represent total mapped divided by total reads × 100 %

### Comparison of four DGE databases

**Fig. 6:**
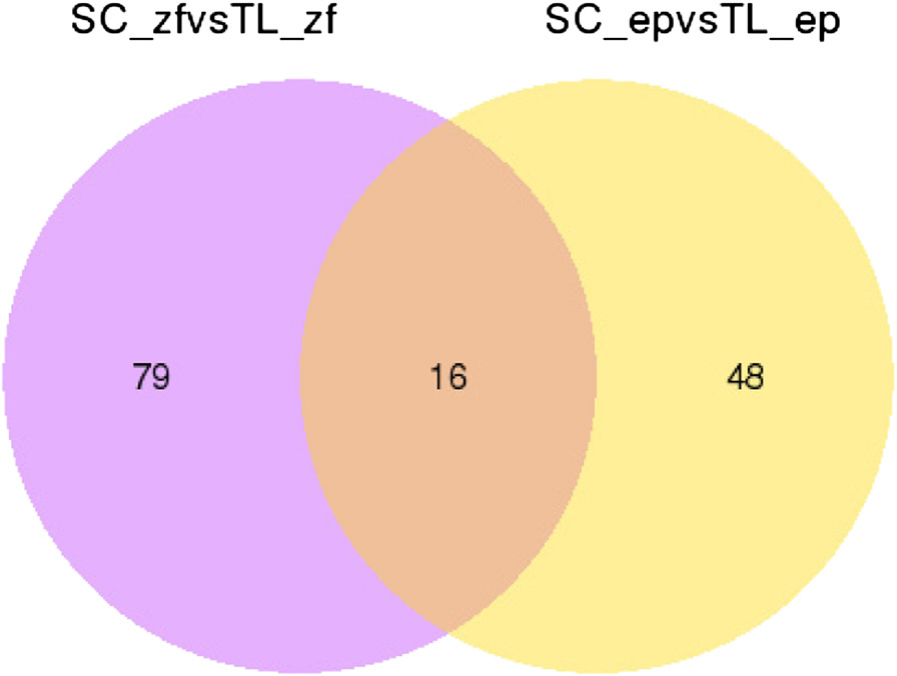
Venn diagram of DEGs from SC_ep vs TL_ep and SC_zf vs TL_zf

We obtained 64 DEGs by comparing the DGE results of SC_ep vs TL_ep. Among the DEGs, 49 were up-regulated and 15 were down-regulated. There were 95 DEGs in SC_zf vs TL_zf, with 71 DEGs being up-regulated and 24 being down-regulated. There were 48 DEGs in SC_ep vs TL_ep and 79 DEGs in SC_zf vs TL_zf. In total, SC_ep vs TL_ep and SC_zf vs TL_zf had 16 DEGs in common (Fig. 6).

### KEGG pathway analysis of four DGE databases

The KEGG database was used to analyze the metabolic pathways of the DEGs of SC_ep vs TL_ep and of SC_zf vs TL_zf. The results showed that 31 DEGs in SC_ep vs TL_ep were enriched in 22 KEGG pathways (Additional file 6). In comparison, 53 DEGs in SC_zf vs TL_zf were enriched in 26 KEGG pathways (Additional file 7). The top four KEGG pathways of SC_ep vs TL_ep were ‘nitrogen metabolism’, ‘cysteine and methionine metabolism’, ‘flavone and flavonol biosynthesis’ and ‘isoquinoline alkaloid biosynthesis’. Regarding up-regulated DEGs, ‘nitrogen metabolism’, ‘flavone and flavonol biosynthesis’, ‘diterpenoid biosynthesis’ and ‘selenocompound metabolism’ were most highly enriched. Regarding down-regulated DEGs, ‘isoquinoline alkaloid biosynthesis’, ‘tropane, piperidine and pyridine alkaloid biosynthesis’, ‘beta-Alanine metabolism’ and ‘carotenoid biosynthesis’ were the four main enriched KEGG pathways. Among the 26 KEGG pathways of SC_zf vs TL_zf, the most enriched pathways were ‘nitrogen metabolism’, ‘alpha-Linolenic acid metabolism’, and ‘glutathione metabolism’. Regarding the up-regulated DEGs, ‘nitrogen metabolism’, and ‘glutathione metabolism’ were mostly highly enriched. For down-regulated DEGs, ‘linoleic acid metabolism’, ‘alpha-linolenic acid metabolism’ and ‘cysteine and methionine metabolism’ were the three main enriched pathways. These results show that calyx persistence in Korla fragrant pear is regulated by a complex transcription mechanism.

We observed that 60 DEGs from SC_ep vs SC_zf, 179 DEGs from TL_ep vs TL_zf, 4 DEGs from SC_ep vs TL_ep, and 3 DEGs from SC_zf vs TL_zf were enriched in the STRING database (http://string-db.org/).

### Real-time quantitative PCR

Ten DEGs were identified by both transcriptome sequencing and DGE sequencing (Table 5). These DEGs included three genes related to plant hormones [ethylene-responsive transcription factor ERF109 OS = *Arabidopsis thaliana* GN = ERF109 PE = 1 SV = 1 (comp36863_c0), ethylene-responsive transcription factor ERF027 OS = *Arabidopsis thaliana* GN = ERF027 PE = 2 SV = 1 (comp44254_c0), auxin-induced protein 5NG4 OS = Pinus taeda PE = 2 SV = 1 (comp50752_c0)]. Three genes were related to cell wall degradation [polygalacturonase inhibitor OS = *Pyrus communis* GN = PGIP PE = 1 SV = 1 (comp49798_c0), beta-galactosidase OS = *Malus domestica* PE = 1 SV = 1 (comp49925_c0), glucan endo-1,3-beta-glucosidase, acidic isoform GI9 OS = *Nicotiana tabacum* GN = PR2 PE = 1 SV = 1 (comp43208_c0)]. Two genes were related to stress [dehydration-responsive protein RD22 [*Prunus persica*] (comp44869_c0), dehydration-responsive element-binding protein, partial [*Malus* × *domestica*] (comp49899_c0)]. One gene was related to lipid transfer protein precursor [*Pisum sativum*] (comp36582_c0), and one gene was involved in NAC domain-containing protein 2 OS = *Arabidopsis thaliana* GN = NAC002 PE = 2 SV = 2 (comp41728_c0). We randomly selected five genes (comp36863_c0, comp41728_c0, com46544_c0, comp49798_c0, comp49614_c0) from the ten DEGs and all of the MYB and SPL genes. The expression levels of these five genes were measured in different floral organs at the early bloom, full bloom, and late bloom stages using qRT-PCR.

**Table 5:** Genes shared by transcriptome and DGE sequencing

Gene Id	Gene description
comp36863_c0	Ethylene-responsive transcription factor ERF109 OS = Arabidopsis thaliana GN = ERF109 PE = 1 SV = 1
comp44254_c0	Ethylene-responsive transcription factor ERF027 OS = Arabidopsis thaliana GN = ERF027 PE = 2 SV = 1
comp50752_c0	Auxin-induced protein 5NG4 OS = Pinus taeda PE = 2 SV = 1
comp49798_c0	Polygalacturonase inhibitor OS = Pyrus communis GN = PGIP PE = 1 SV = 1
comp49925_c0	Beta-galactosidase OS = Malus domestica PE = 1 SV = 1
comp43208_c0	Glucan endo-1,3-beta-glucosidase, acidic isoform GI9 OS = Nicotiana tabacum GN = PR2 PE = 1 SV = 1
comp44869_c0	Dehydration-responsive protein RD22 OS = Arabidopsis thaliana GN = RD22 PE = 2 SV = 1
comp49899_c0	Dehydration-responsive element-binding protein 1A OS = Arabidopsis thaliana GN = DREB1A PE = 1 SV = 2
comp36582_c0	Non-specific lipid-transfer protein OS = Pyrus communis PE = 1 SV = 1
comp41728_c0	NAC domain-containing protein 2 OS = Arabidopsis thaliana GN = NAC002 PE = 2 SV = 2

**Fig. 7:**
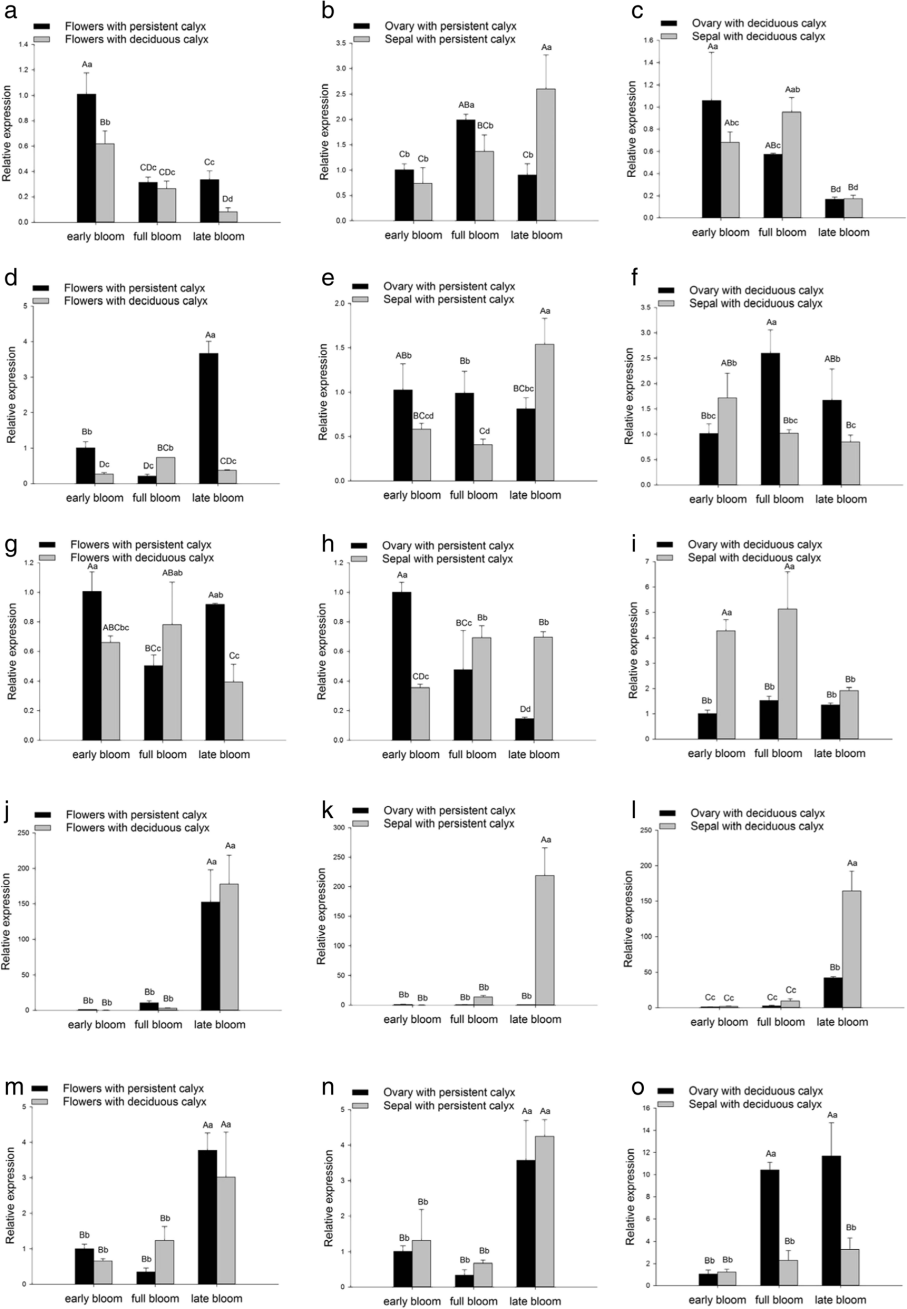
Temporal changes in the expression of selected genes in complete flowers, ovaries, and sepals. Error bars indicate SD. Different lowercase letters within a panel indicate significant differences at *P* = 0.05. Different uppercase letters within a panel indicate significant differences at *P* = 0.01

The expression of ERF109 at the early bloom and late bloom stages was significantly (*P* = 0.01) greater in flowers with persistent calyx than in flowers with deciduous calyx. Regardless of whether the flower had a deciduous or a persistent calyx, ERF109 expression was significantly (*P* = 0.01) greater at the early bloom stage than at either the full bloom or late bloom stages (Fig. 7a). The expression of ERF109 at the late bloom stage was significantly (*P* = 0.01) greater in ovaries with persistent calyx than in sepals with persistent calyx (Fig. 7b). Regardless of bloom stage, the expression of ERF109 in ovaries with deciduous calyx was not significantly different than that in sepals with deciduous calyx (Fig. 7c).

The expression of NAC002 in flowers varied significantly depending on the type of calyx and the flower stage. Specifically, NAC002 expression at early bloom and late bloom was significantly (*P* = 0.01) greater in flowers with persistent calyx than in flowers with deciduous calyx; however, the opposite was observed at full bloom (Fig. 7d). The NAC002 expression in flowers with a persistent calyx was significantly (*P* = 0.01) highest at the late bloom and early bloom stages. In contrast, NAC002 expression in flowers with a deciduous calyx was significantly (*P* = 0.05) greatest at the full bloom stage. The expression of NAC002 in ovaries with persistent calyx was significantly greater than that in sepals with persistent calyx at the early bloom stage (*P* = 0.05) and at the full bloom stage (*P* = 0.01) (Fig. 7e). In contrast, at the late bloom stage, NAC002 expression in ovaries with persistent calyx was significantly (*P* = 0.01) less than that in sepals with persistent calyx. The expression of NAC002 in ovaries with deciduous calyx was significantly greater than that in sepals with deciduous calyx at the full bloom (*P* = 0.01) and late bloom stages (*P* = 0.05) (Fig. 7f).

The expression of MYB5 was significantly greater in flowers with persistent calyx than in flowers with deciduous calyx at the early bloom (*P* = 0.05) and late bloom (*P* = 0.01) stages (Fig. 7g). In contrast, at the full bloom stage, MYB5 expression was significantly (*P* = 0.05) less in flowers with persistent calyx than in flowers with deciduous calyx. The expression of MYB5 in sepals with persistent calyx was significantly greater than that in ovaries with persistent calyx at the full bloom (*P* = 0.05) and late bloom (*P* = 0.01) stages (Fig. 7h). In contrast, MYB5 expression at the early bloom stage was significantly (*P* = 0.01) less in sepals with persistent calyx than in ovaries with persistent calyx. The expression of MYB5 in sepals with deciduous calyx was significantly greater than that in ovaries with deciduous calyx at early bloom and full bloom (Fig. 7i, *P* = 0.01).

Regardless of whether the flower had a deciduous or a persistent calyx, PGIG expression was significantly (*P* = 0.01) greater at the late bloom stage than at either the early bloom or full bloom stages (Fig. 7j). There was no significant difference in PGIG expression between flowers with persistent calyx and flowers with deciduous calyx. Regardless of whether the calyx was persistent or deciduous, the expression of PGIG in sepals was significantly greater than that in ovaries at the late bloom stage (Fig. 7k and l, *P* = 0.01).

The expression of SPL9 at the early bloom and late bloom stages was greater in flowers with persistent calyx than in flowers with deciduous calyx; however the opposite was true at the full bloom stage. The expression of SPL9 in flowers with deciduous calyx was not significantly different from that in flowers with deciduous calyx. Regardless of whether the flower had a deciduous or a persistent calyx, SPL9 expression was significantly (*P* = 0.01) greater at the late bloom stage than at either the early bloom or full bloom stages (Fig. 7m). There was no significant difference in SPL9 expression between ovaries with persistent calyx and sepals with deciduous calyx (Fig. 7n). The expression of MYB5 in ovaries with deciduous calyx was significantly greater than that in sepals at the full bloom and late bloom stages (Fig. 7o, *P* = 0.01).

The total expression pattern of the three genes ((ERF109 (comp36863_c0), NAC002 (comp41728_c0), and PGIG (comp49798_c0)) obtained with qRT-PCR was consistent with the RNA-seq data. This confirmed the validity of our results.

### Plant hormone and organ abscission

Many hormones, especially IAA and ethylene, regulate organ abscission [30–35]. From 103 DEGs in SC_hua vs TL_hua, 11 genes were identified that were related to plant hormone metabolism. Five of these genes were related to ethylene-responsive transcription factor, two genes were related to auxin-induced protein, one gene was related to gibberellin-regulated protein, one gene was related to EREBP-like factor, one gene was related to the auxin responsive GH3 gene family, and one gene was related to brassinosteroid-regulated protein. From 64 DEGs in SC_ep vs TL_ep, seven genes were identified that were involved in plant hormone metabolism. Four of these genes were related to ethylene-responsive transcription factor, one gene was related to gibberellin 2-beta-dioxygenase 1, one gene was related to auxin-induced protein, and one gene was related to abscisic acid 8'-hydroxylase 4. We also identified five genes related to ethylene-responsive transcription factor from 95 DEGs in SC_zf vs TL_zf (Table 6).

**Table 6:** Genes related to plant hormones

Gene Id	Gene description
SC-hua vs TL-hua	
comp33730_c0	Gibberellin-regulated protein 14 OS = Arabidopsis thaliana GN = GASA14 PE = 1 SV = 1
comp36863_c0	Ethylene-responsive transcription factor ERF109 OS = Arabidopsis thaliana GN = ERF109 PE = 1 SV = 1
comp43830_c0	Auxin-induced protein 5NG4 OS = Pinus taeda PE = 2 SV = 1
comp44254_c0	Ethylene-responsive transcription factor ERF027 OS = Arabidopsis thaliana GN = ERF027 PE = 2 SV = 1
comp44440_c0	Ethylene-responsive transcription factor ERF109 OS = Arabidopsis thaliana GN = ERF109 PE = 1 SV = 1
comp47703_c0	Ethylene-responsive transcription factor CRF4 OS = Arabidopsis thaliana GN = CRF4 PE = 1 SV = 2
comp50752_c0	Auxin-induced protein 5NG4 OS = Pinus taeda PE = 2 SV = 1
comp54623_c0	Ethylene-responsive transcription factor ERF109 OS = Arabidopsis thaliana GN = ERF109 PE = 1 SV = 1
comp39099_c0	EREBP-like factor
comp50238_c0	auxin responsive GH3 gene family
comp49181_c2	Brassinosteroid-regulated protein BRU1 OS = Glycine max PE = 2 SV = 1
SC_ep vs TL_ep	
comp33823_c0	Gibberellin 2-beta-dioxygenase 1 OS = Pisum sativum GN = GA2OX1 PE = 1 SV = 1
comp36863_c0	Ethylene-responsive transcription factor ERF109 OS = Arabidopsis thaliana GN = ERF109 PE = 1 SV = 1
comp43552_c0	Ethylene-responsive transcription factor RAP2-4 OS = Arabidopsis thaliana GN = RAP2-4 PE = 1 SV = 1
comp44254_c0	Ethylene-responsive transcription factor ERF027 OS = Arabidopsis thaliana GN = ERF027 PE = 2 SV = 1
comp50299_c0	Abscisic acid 8’-hydroxylase 4 OS = Arabidopsis thaliana GN = CYP707A4 PE = 2 SV = 2
comp50752_c0	Auxin-induced protein 5NG4 OS = Pinus taeda PE = 2 SV = 1
comp48588_c0	Ethylene-responsive transcription factor 1A OS = Arabidopsis thaliana GN = ERF1A PE = 1 SV = 2
SC_zf vs TL_zf	
comp36863_c0	Ethylene-responsive transcription factor ERF109 OS = Arabidopsis thaliana GN = ERF109 PE = 1 SV = 1
comp40246_c0	Ethylene-responsive transcription factor ERF023 OS = Arabidopsis thaliana GN = ERF023 PE = 2 SV = 1
comp41236_c0	Ethylene-responsive transcription factor ERF019 OS = Arabidopsis thaliana GN = ERF019 PE = 2 SV = 1
comp43552_c0	Ethylene-responsive transcription factor RAP2-4 OS = Arabidopsis thaliana GN = RAP2-4 PE = 1 SV = 1
comp47393_c0	Ethylene-responsive transcription factor ERF105 OS = Arabidopsis thaliana GN = ERF105 PE = 2 SV = 1

### Genes related to cell wall degradation and organ abscission

The dissolution of the middle lamella is related to abscission, especially the loss of adhesion by separation layer cells due to the effects of cell wall degrading enzymes such as polygalacturonases. Several researchers have reported that cell wall modifying proteins such as expansin [36] and pectinesterase [37] have a role in abscission. Other researchers have observed that polygalacturonases have important function in the abscission process in oil palm [38], tomato [39], oilseed rape and Arabidopsis [40]. Beta-galactosidase [41], xyloglucan endotransglucosylase/hydrolase [42], and glucanase [43] genes have also been shown to be related to abscission. We obtained eight genes related to cell wall degradation from DEGs in SC_hua vs TL_hua. These eight genes included one gene related to polygalacturonase, one gene related to polygalacturonase inhibition, one gene related to beta-galactosidase, one gene related to glucan endo-1,3-beta-glucosidase, one gene related to lignin catabolic process, one gene related to tissue regeneration, and two genes related to xyloglucan endotransglucosylase. One expansin gene was obtained from DEGs in SC_ep vs TL_ep. From DEGs in SC_zf vs TL_zf, we obtained genes related to glucan endo-1,3-beta-glucosidase, beta-galactosidase, polygalacturonase inhibition, xyloglucan endotransglucosylase, and pectinesterase (Table 7).

**Table 7:** Genes related to cell wall metabolism

Gene Id	Gene description
SC-hua vs TL-hua	
comp47965_c0	Probable polygalacturonase OS = Vitis vinifera GN = GSVIVT00026920001 PE = 1 SV = 1
comp49798_c0	Polygalacturonase inhibitor OS = Pyrus communis GN = PGIP PE = 1 SV = 1
comp49925_c0	Beta-galactosidase OS = Malus domestica PE = 1 SV = 1
comp43208_c0	Glucan endo-1,3-beta-glucosidase, acidic isoform GI9 OS = Nicotiana tabacum GN = PR2 PE = 1 SV = 1
comp40498_c0	lignin catabolic process//oxidation-reduction process
comp45343_c2	tissue regeneration//cell adhesion//regulation of transcription, DNA-dependent
comp49181_c2	Malus x domestica xyloglucan endotransglucosylase/hydrolase 7 mRNA, complete cds
comp38937_c0	Probable xyloglucan endotransglucosylase/hydrolase protein 23 OS = Arabidopsis thaliana GN = XTH23 PE = 2 SV = 1
SC_ep vs TL_ep	
comp43526_c0	Expansin-A8 OS = Arabidopsis thaliana GN = EXPA8 PE = 2 SV = 1
SC_zf vs TL_zf	
comp45273_c0	Glucan endo-1,3-beta-glucosidase 8 OS = Arabidopsis thaliana GN = At1g64760 PE = 1 SV = 2
comp49925_c0	Beta-galactosidase OS = Malus domestica PE = 1 SV = 1
comp43208_c0	Glucan endo-1,3-beta-glucosidase, acidic isoform GI9 OS = Nicotiana tabacum GN = PR2 PE = 1 SV = 1
comp49798_c0	Polygalacturonase inhibitor OS = Pyrus communis GN = PGIP PE = 1 SV = 1
comp38937_c0	Probable xyloglucan endotransglucosylase/hydrolase protein 23 OS = Arabidopsis thaliana GN = XTH23 PE = 2 SV = 1
comp51877_c0	Putative pectinesterase/pectinesterase inhibitor 28 OS = Arabidopsis thaliana GN = PME28 PE = 2 SV = 1

### Function of SPL and MYB genes in organ abscission

The SPL genes play an important role in the growth process of plants, including morphogenesis, the transition between developmental stages, sporogenesis, floral and fruit development, stress response, and plant hormone signal transduction [44]. In addition, SPL genes are induced during cell senescence leading to cell death [45, 46]. The MYB genes participate in plant secondary metabolism [47] as well as the plant’s response to hormones and environmental factors [48–50]. The MYB genes also regulate cellular differentiation, the cell life cycle [51, 52], and the morphogenesis of organs such as leaves [53–55]. The MYB genes are also involved in abscission [11, 56, 46]. We obtained 98 MYB and 21 SPL genes from the 48894 annotated unigenes (Table 8).

**Table 8:** The MYB and SPL genes

Gene ID	Gene description
SPL	
comp40233_c0	Squamosa promoter-binding-like protein 8 OS = Arabidopsis thaliana GN = SPL8 PE = 1 SV = 2
comp36894_c0	Squamosa promoter-binding-like protein 13B OS = Arabidopsis thaliana GN = SPL13B PE = 3 SV = 1
comp54049_c0	Squamosa promoter-binding-like protein 14 OS = Arabidopsis thaliana GN = SPL14 PE = 2 SV = 3
comp15760_c0	Squamosa promoter-binding-like protein 5 OS = Arabidopsis thaliana GN = SPL5 PE = 2 SV = 1
comp53959_c0	Squamosa promoter-binding-like protein 1 OS = Arabidopsis thaliana GN = SPL1 PE = 1 SV = 2
comp48948_c0	Squamosa promoter-binding-like protein 1 OS = Arabidopsis thaliana GN = SPL1 PE = 1 SV = 2
comp51995_c0	Squamosa promoter-binding-like protein 6 OS = Arabidopsis thaliana GN = SPL6 PE = 2 SV = 2
comp43799_c2	Squamosa promoter-binding-like protein 12 OS = Arabidopsis thaliana GN = SPL12 PE = 1 SV = 1
comp33051_c0	Putative squamosa promoter-binding-like protein 19 OS = Oryza sativa subsp. japonica GN = SPL19 PE = 3 SV = 2
comp43799_c1	Squamosa promoter-binding-like protein 1 OS = Arabidopsis thaliana GN = SPL1 PE = 1 SV = 2
comp19424_c0	Squamosa promoter-binding-like protein 16 OS = Arabidopsis thaliana GN = SPL16 PE = 2 SV = 2
comp43328_c0	Squamosa promoter-binding-like protein 4 OS = Arabidopsis thaliana GN = SPL4 PE = 1 SV = 1
comp34651_c0	Malus x domestica SPL domain class transcription factor (SPL3) mRNA, complete cds
comp48364_c1	Squamosa promoter-binding-like protein 12 OS = Oryza sativa subsp. indica GN = SPL12 PE = 2 SV = 1
comp30499_c0	Malus x domestica SPL domain class transcription factor (SPL2) mRNA, complete cds
comp46477_c1	Squamosa promoter-binding-like protein 7 OS = Oryza sativa subsp. japonica GN = SPL7 PE = 2 SV = 2
comp49614_c0	Squamosa promoter-binding-like protein 9 OS = Arabidopsis thaliana GN = SPL9 PE = 2 SV = 2
comp53802_c0	Squamosa promoter-binding-like protein 7 OS = Arabidopsis thaliana GN = SPL7 PE = 1 SV = 2
comp47538_c0	Squamosa promoter-binding-like protein 6 OS = Arabidopsis thaliana GN = SPL6 PE = 2 SV = 2
comp48561_c0	Squamosa promoter-binding-like protein 8 OS = Arabidopsis thaliana GN = SPL8 PE = 1 SV = 2
comp17109_c0	Malus x domestica SPL domain class transcription factor (SPL3) mRNA, complete cds
MYB	
comp491996_c0	putative MYB transcription factor [Rosa rugosa]
comp47342_c0	Myb-related protein 308 OS = Antirrhinum majus GN = MYB308 PE = 2 SV = 1
comp47241_c0	Myb-related protein 306 OS = Antirrhinum majus GN = MYB306 PE = 2 SV = 1
comp45253_c0	Anthocyanin regulatory C1 protein OS = Zea mays GN = C1 PE = 2 SV = 1
comp44151_c0	Protein ODORANT1 OS = Petunia hybrida GN = ODO1 PE = 2 SV = 1
comp31710_c0	Transcription factor MYB39 OS = Arabidopsis thaliana GN = MYB39 PE = 2 SV = 1
comp42545_c0	Transcription factor RAX3 OS = Arabidopsis thaliana GN = RAX3 PE = 2 SV = 1
comp41210_c0	Myb-related protein 3R-1 OS = Arabidopsis thaliana GN = MYB3R-1 PE = 2 SV = 1
comp2739_c0	Myb-related protein Myb4 OS = Oryza sativa subsp. japonica GN = MYB4 PE = 2 SV = 2
comp23664_c0	Myb-related protein 306 OS = Antirrhinum majus GN = MYB306 PE = 2 SV = 1
comp49924_c0	Transcription factor MYB1R1 OS = Solanum tuberosum PE = 2 SV = 1
comp47011_c1	Transcription factor MYB86 OS = Arabidopsis thaliana GN = MYB86 PE = 2 SV = 1
comp45831_c0	Transcription repressor MYB6 OS = Arabidopsis thaliana GN = MYB6 PE = 1 SV = 1
comp259366_c0	Malus x domestica MYBR domain class transcription factor (MYBR14) mRNA, complete cds
comp25899_c0	Malus x domestica MYB domain class transcription factor (MYB31) mRNA, complete cds
comp51661_c2	Malus x domestica MYB domain class transcription factor (MYB88) mRNA, complete cds
comp38641_c1	Anthocyanin regulatory C1 protein OS = Zea mays GN = C1 PE = 2 SV = 1
comp36088_c0	Transcription factor MYB113 OS = Arabidopsis thaliana GN = MYB113 PE = 1 SV = 1
comp33026_c0	Transcription factor MYB3 OS = Arabidopsis thaliana GN = MYB3 PE = 1 SV = 1
comp44651_c0	Myb-related protein 306 OS = Antirrhinum majus GN = MYB306 PE = 2 SV = 1
comp41277_c0	Malus x domestica MYB92 mRNA, complete cds
comp42019_c0	Transcription factor MYB21 OS = Arabidopsis thaliana GN = MYB21 PE = 1 SV = 1
comp42660_c0	Transcription factor MYB39 OS = Arabidopsis thaliana GN = MYB39 PE = 2 SV = 1
comp617_c1	Pyrus communis R2R3 MYB transcription factor 10 (MYB10) gene, promoter region and partial cds
comp266782_c0	MYB11 [Malus x domestica]
comp5228_c0	Transcription factor MYB82 OS = Arabidopsis thaliana GN = MYB82 PE = 1 SV = 1
comp40270_c0	Transcription repressor MYB4 OS = Arabidopsis thaliana GN = MYB4 PE = 1 SV = 1
comp41339_c0	Malus x domestica MYB7 mRNA, complete cds
comp40714_c1	MYB92 [Malus x domestica]
comp44744_c0	Transcription factor MYB44 OS = Arabidopsis thaliana GN = MYB44 PE = 2 SV = 1
comp38255_c0	Malus x domestica cultivar Royal Gala MYB10 (MYB10) gene, promoter region and complete cds
comp33193_c0	Malus x domestica MYB domain class transcription factor (MYB33) mRNA, complete cds
comp411_c0	PREDICTED: Cicer arietinum transcription factor MYB12-like (LOC101507446), Mrna
comp33184_c0	Malus x domestica MYB domain class transcription factor (MYB33) mRNA, complete cds
comp38919_c0	MYB24 [Malus x domestica]
comp9080_c0	PREDICTED: Fragaria vesca subsp. vesca transcription factor MYB32-like (LOC101307403), mRNA
comp37971_c0	Myb-related protein 305 OS = Antirrhinum majus GN = MYB305 PE = 2 SV = 1
comp8954_c0	Malus x domestica MYB domain class transcription factor (MYB36) mRNA, complete cds
comp52545_c0	Malus x domestica MYBR domain class transcription factor (MYBR8) mRNA, complete cds
comp51661_c0	Myb-related protein B OS = Xenopus laevis GN = mybl2 PE = 2 SV = 2
comp28973_c0	Malus x domestica MYB domain class transcription factor (MYB1) mRNA, complete cds
comp404278_c0	Transcription factor MYB39 OS = Arabidopsis thaliana GN = MYB39 PE = 2 SV = 1
comp44434_c0	Transcription factor MYB12 OS = Arabidopsis thaliana GN = MYB12 PE = 2 SV = 1
comp40714_c0	MYB92 [Malus x domestica]
comp620621_c0	Transcription factor MYB23 OS = Arabidopsis thaliana GN = MYB23 PE = 1 SV = 1
comp23111_c0	Myb-related protein Myb4 OS = Oryza sativa subsp. japonica GN = MYB4 PE = 2 SV = 2
comp43823_c0	Myb-related protein Myb4 OS = Oryza sativa subsp. japonica GN = MYB4 PE = 2 SV = 2
comp44151_c1	MYB19 [Malus x domestica] > gi|189339113|dbj|BAG48172.1| myb-related transcription factor [Malus x domestica]
comp49161_c0	Transcription factor MYB44 OS = Arabidopsis thaliana GN = MYB44 PE = 2 SV = 1
comp49501_c0	Transcription factor MYB1R1 OS = Solanum tuberosum PE = 2 SV = 1
comp48408_c0	Transcription factor MYB44 OS = Arabidopsis thaliana GN = MYB44 PE = 2 SV = 1
comp35657_c0	Transcription factor MYB48 OS = Arabidopsis thaliana GN = MYB48 PE = 2 SV = 1
comp46544_c0	Transcription repressor MYB5 OS = Arabidopsis thaliana GN = MYB5 PE = 1 SV = 1
comp7072_c1	Myb-related protein 306 OS = Antirrhinum majus GN = MYB306 PE = 2 SV = 1
comp46515_c0	Transcription factor MYB86 OS = Arabidopsis thaliana GN = MYB86 PE = 2 SV = 1
comp30457_c0	Malus x domestica MYB domain class transcription factor (MYB25) mRNA, complete cds
comp49893_c0	Transcription factor MYB44 OS = Arabidopsis thaliana GN = MYB44 PE = 2 SV = 1
comp46778_c0	Myb-related protein 305 OS = Antirrhinum majus GN = MYB305 PE = 2 SV = 1
comp89753_c0	Transcription factor MYB12 OS = Arabidopsis thaliana GN = MYB12 PE = 2 SV = 1
comp44726_c0	Transcription factor AS1 OS = Arabidopsis thaliana GN = AS1 PE = 1 SV = 1
comp25436_c0	Transcription factor MYB44 OS = Arabidopsis thaliana GN = MYB44 PE = 2 SV = 1
comp27942_c0	Transcription factor MYB44 OS = Arabidopsis thaliana GN = MYB44 PE = 2 SV = 1
comp46739_c0	Transcription factor MYB44 OS = Arabidopsis thaliana GN = MYB44 PE = 2 SV = 1
comp40636_c1	Transcription factor MYB113 OS = Arabidopsis thaliana GN = MYB113 PE = 1 SV = 1
comp38343_c0	Transcription factor MYB3 OS = Arabidopsis thaliana GN = MYB3 PE = 1 SV = 1
comp41103_c0	Transcription repressor MYB5 OS = Arabidopsis thaliana GN = MYB5 PE = 1 SV = 1
comp33109_c1	Rosa rugosa mRNA for putative MYB transcription factor (myb9 gene)
comp209723_c0	putative MYB transcription factor [Rosa hybrid cultivar]
comp44561_c0	Myb-related protein Myb4 OS = Oryza sativa subsp. japonica GN = MYB4 PE = 2 SV = 2
comp51083_c0	putative MYB transcription factor [Rosa hybrid cultivar]
comp31372_c0	MYB domain class transcription factor [Malus x domestica]
comp38343_c1	Myb-related protein Myb4 OS = Oryza sativa subsp. japonica GN = MYB4 PE = 2 SV = 2
comp52029_c2	Malus x domestica cultivar Jiangsu Fuji MYB transcription factor (MYB53) mRNA, partial cds
comp45889_c1	Transcription factor MYB39 OS = Arabidopsis thaliana GN = MYB39 PE = 2 SV = 1
comp37277_c1	Transcription factor MYB59 OS = Arabidopsis thaliana GN = MYB59 PE = 2 SV = 2
comp46917_c0	Myb-related protein 330 OS = Antirrhinum majus GN = MYB330 PE = 2 SV = 1
comp7908_c0	Transcription factor MYB86 OS = Arabidopsis thaliana GN = MYB86 PE = 2 SV = 1
comp7072_c0	Transcription factor MYB39 OS = Arabidopsis thaliana GN = MYB39 PE = 2 SV = 1
comp47011_c0	Myb-related protein Hv33 OS = Hordeum vulgare GN = MYB2 PE = 2 SV = 3
comp159049_c0	Transcription factor MYB46 OS = Arabidopsis thaliana GN = MYB46 PE = 2 SV = 1
comp27400_c0	Transcription factor MYB46 OS = Arabidopsis thaliana GN = MYB46 PE = 2 SV = 1
comp308054_c0	Malus x domestica MYB domain class transcription factor (MYB18) mRNA, complete cds
comp125091_c0	Malus x domestica cultivar Royal Gala MYB9 mRNA, complete cds
comp48140_c0	Rosa hybrid cultivar mRNA for putative MYB transcription factor (myb1 gene), cultivar Yellow Island
comp8463_c0	Transcription factor MYB44 OS = Arabidopsis thaliana GN = MYB44 PE = 2 SV = 1
comp26540_c0	Malus x domestica cultivar Royal Gala MYB9 mRNA, complete cds
comp28178_c0	Malus x domestica MYB2 mRNA, complete cds
comp188108_c0	Transcription factor MYB113 OS = Arabidopsis thaliana GN = MYB113 PE = 1 SV = 1
comp29648_c0	Myb-related protein 305 OS = Antirrhinum majus GN = MYB305 PE = 2 SV = 1
comp611736_c0	Malus x domestica MYB domain class transcription factor (MYB17) mRNA, complete cds
comp49971_c0	Transcription factor MYB86 OS = Arabidopsis thaliana GN = MYB86 PE = 2 SV = 1
comp42161_c1	Lupinus albus LaMYB27 mRNA for R2R3-MYB transcription factor, partial cds
comp43170_c0	Transcription factor MYB21 OS = Arabidopsis thaliana GN = MYB21 PE = 1 SV = 1
comp37565_c0	Trifolium repens tannin-related R2R3 MYB transcription factor (Myb14) gene, Myb14-3 allele, partial cds
comp49430_c0	Myb-related protein 3R-1 OS = Arabidopsis thaliana GN = MYB3R-1 PE = 2 SV = 1
comp43202_c0	Myb-related protein 305 OS = Antirrhinum majus GN = MYB305 PE = 2 SV = 1
comp38641_c0	Malus x domestica MYB11 mRNA, complete cds
comp50379_c0	Myb-related protein 305 OS = Antirrhinum majus GN = MYB305 PE = 2 SV = 1

### Stress response genes and abscission

The sequencing results showed that many genes related to stress response exhibited differential expression. There was one heat shock factor protein, two dehydration-responsive element-binding proteins, one dehydration-responsive protein, two NAC transcription factor proteins, one NAC domain-containing protein [57, 58], and one cysteine synthase-like gene [59] among the DEGs in SC_hua vs TL_hua. There were also genes related to the NAC domain-containing protein, the pathogenesis-related protein Bet v I family, the senescence-related protein gene, dehydration-responsive protein, and dehydration-responsive element-binding protein from DEGs in SC_ep vs TL_ep. From the DEGs in SC_zf vs TL_zf, we obtained genes related to disease resistance response protein 206, dehydration-responsive protein, defensin-like protein, and senescence-related protein (Table 9).

**Table 9:** Genes related to stress

Gene Id	Gene description
SC-hua vs TL-hua	
comp43473_c0	Heat shock factor protein HSF24 OS = Solanum peruvianum GN = HSF24 PE = 2 SV = 1
comp44869_c0	Dehydration-responsive protein RD22 OS = Arabidopsis thaliana GN = RD22 PE = 2 SV = 1
comp49899_c0	Dehydration-responsive element-binding protein 1A OS = Arabidopsis thaliana GN = DREB1A PE = 1 SV = 2
comp39099_c0	Dehydration-responsive element-binding protein 3 OS = Arabidopsis thaliana GN = DREB3 PE = 2 SV = 1
comp45992_c0	NAC transcription factor NAM-B2 OS = Triticum durum GN = NAM-B2 PE = 2 SV = 1
comp49969_c0	NAC transcription factor 25 OS = Arabidopsis thaliana GN = NAC025 PE = 2 SV = 1
comp41728_c0	NAC domain-containing protein 2 OS = Arabidopsis thaliana GN = NAC002 PE = 2 SV = 2
comp48683_c0	Cysteine synthase OS = Citrullus lanatus PE = 1 SV = 1
SC_ep vs TL_ep	
comp34503_c0	Pathogenesis-related protein Bet v I family
comp43933_c0	senescence-related protein [Camellia sinensis]
comp44869_c0	Dehydration-responsive protein RD22 OS = Arabidopsis thaliana GN = RD22 PE = 2 SV = 1
comp41728_c0	NAC domain-containing protein 2 OS = Arabidopsis thaliana GN = NAC002 PE = 2 SV = 2
comp49899_c0	Dehydration-responsive element-binding protein 1A OS = Arabidopsis thaliana GN = DREB1A PE = 1 SV = 2
SC_zf vs TL_zf	
comp41222_c0	Disease resistance response protein 206 OS = Pisum sativum GN = PI206 PE = 2 SV = 2
comp44869_c0	Dehydration-responsive protein RD22 OS = Arabidopsis thaliana GN = RD22 PE = 2 SV = 1
comp51764_c0	Defensin-like protein 2 OS = Arabidopsis thaliana GN = PDF2.2 PE = 2 SV = 1
comp43933_c0	senescence-related protein [Camellia sinensis]

### Other genes and abscission

Several researchers have reported that zinc finger protein [60] and lipid-transfer protein [61, 62] are involved in calyx abscission. We obtained one gene related to lipid-transfer protein from DEGs in SC_hua vs TL_hua. One gene related to lipid-transfer protein as well as five zinc finger genes were obtained from DEGs in SC_zf vs TL_zf (Table 10).

**Table 10:** Additional genes related to abscission

Gene Id	Gene description
SC-hua vs TL-hua	
comp36582_c0	Non-specific lipid-transfer protein OS = Pyrus communis PE = 1 SV = 1
SC_zf vs TL_zf	
comp33569_c0	zinc finger protein, putative [Ricinus communis] > gi|223538542|gb|EEF40147.1| zinc finger protein, putative [Ricinus communis]
comp41672_c0	Zinc finger, C3HC4 type (RING finger)//Ring finger domain//Anaphase-promoting complex subunit 11 RING-H2 finger//zinc-RING finger domain//RING-H2 zinc finger
comp43820_c0	MYM-type Zinc finger with FCS sequence motif
comp46839_c0	Putative zinc finger protein At1g68190 OS = Arabidopsis thaliana GN = At1g68190 PE = 2 SV = 1
comp53961_c0	RING finger and CHY zinc finger domain-containing protein 1 OS = Homo sapiens GN = RCHY1 PE = 1 SV = 1
comp36582_c0	Non-specific lipid-transfer protein OS = Pyrus communis PE = 1 SV = 1

### Putative genes related to abscission

Other genes in this study showed high-level differential expression. However, the function of these genes is unknown. We defined these genes as putative genes related to abscission. There were ten putative genes among DEGs in SC_hua vs TL_hua, eleven putative genes among DEGs in SC_ep vs TL_ep, and eighteen putative genes among DEGs in SC_zf vs TL_zf (Table 11).

**Table 11:** Putative genes related to abscission

Gene Id	Gene description
SC-hua vs TL-hua	
comp54231_c0	Polyphenol oxidase, chloroplastic OS = Malus domestica PE = 2 SV = 1
comp52712_c0	Asparagine synthetase [glutamine-hydrolyzing] OS = Asparagus officinalis PE = 2 SV = 2
comp48325_c1	NADP-dependent D-sorbitol-6-phosphate dehydrogenase OS = Malus domestica GN = S6PDH PE = 2 SV = 1
comp33824_c0	11S globulin seed storage protein 2 OS = Sesamum indicum PE = 2 SV = 1
comp42796_c0	CASP-like protein RCOM_0679870 OS = Ricinus communis GN = RCOM_0679870 PE = 2 SV = 1
comp44393_c0	UDP-glucose 4-epimerase 1 OS = Arabidopsis thaliana GN = At1g12780 PE = 1 SV = 2
comp32282_c0	Alpha-aminoadipic semialdehyde synthase OS = Arabidopsis thaliana GN = LKR/SDH PE = 1 SV = 1
comp44627_c0	Protein ASPARTIC PROTEASE IN GUARD CELL 1 OS = Arabidopsis thaliana GN = ASPG1 PE = 1 SV = 1
comp53838_c1	Synaptotagmin-3 OS = Arabidopsis thaliana GN = SYT3 PE = 2 SV = 1
comp46366_c0	Tonoplast dicarboxylate transporter OS = Arabidopsis thaliana GN = TDT PE = 2 SV = 2
SC_ep vs TL_ep	
comp47776_c0	Bidirectional sugar transporter NEC1 OS = Petunia hybrida GN = NEC1 PE = 2 SV = 1
comp43067_c0	Miraculin OS = Richadella dulcifica PE = 1 SV = 3
comp44995_c0	Taxadien-5-alpha-ol O-acetyltransferase OS = Taxus wallichiana var. chinensis PE = 2 SV = 1
comp43847_c0	Uncharacterized protein At3g61260 OS = Arabidopsis thaliana GN = At3g61260 PE = 1 SV = 1
comp47017_c0	PREDICTED: Fragaria vesca subsp. vesca uncharacterized LOC101305493 (LOC101305493), mRNA
comp43990_c0	Heavy metal-associated isoprenylated plant protein 26 OS = Arabidopsis thaliana GN = HIPP26 PE = 1 SV = 1
comp47206_c0	Copper methylamine oxidase OS = Arthrobacter sp. (strain P1) GN = maoII PE = 1 SV = 1
comp51202_c0	Diacylglycerol kinase 1 OS = Arabidopsis thaliana GN = DGK1 PE = 1 SV = 2
comp50351_c0	Uncharacterized membrane protein C2G11.09 OS = Schizosaccharomyces pombe (strain 972/ATCC 24843) GN = SPAC2G11.09 PE = 2 SV = 2
comp44384_c1	LOB domain-containing protein 41 OS = Arabidopsis thaliana GN = LBD41 PE = 2 SV = 1
comp46169_c0	--
SC_zf vs TL_zf	
comp40401_c0	Jasmonate O-methyltransferase OS = Brassica rapa subsp. pekinensis GN = JMT PE = 1 SV = 1
comp42909_c0	Ornithine decarboxylase OS = Datura stramonium PE = 2 SV = 1
comp40364_c1	Bifunctional monodehydroascorbate reductase and carbonic anhydrase nectarin-3 OS = Nicotiana langsdorffii x Nicotiana sanderae GN = NEC3 PE = 1 SV = 1
comp49118_c1	Polyphenol oxidase, chloroplastic OS = Malus domestica PE = 2 SV = 1
comp48325_c1	NADP-dependent D-sorbitol-6-phosphate dehydrogenase OS = Malus domestica GN = S6PDH PE = 2 SV = 1
comp48520_c0	Beta-D-xylosidase 1 OS = Arabidopsis thaliana GN = BXL1 PE = 1 SV = 1
comp36479_c0	--
comp44627_c0	Protein ASPARTIC PROTEASE IN GUARD CELL 1 OS = Arabidopsis thaliana GN = ASPG1 PE = 1 SV = 1
comp54257_c0	Beta-fructofuranosidase, insoluble isoenzyme 1 OS = Daucus carota GN = INV1 PE = 1 SV = 1
comp26144_c0	(RS)-norcoclaurine 6-O-methyltransferase OS = Coptis japonica PE = 1 SV = 1
comp47734_c0	(R)-mandelonitrile lyase 3 OS = Prunus serotina GN = MDL3 PE = 2 SV = 1
comp45349_c1	Snakin-2 OS = Solanum tuberosum GN = SN2 PE = 1 SV = 1
comp47553_c0	Ferredoxin--nitrite reductase, chloroplastic OS = Betula pendula GN = NIR1 PE = 2 SV = 1
comp42868_c0	L-aspartate oxidase 1 OS = Ralstonia solanacearum (strain GMI1000) GN = nadB1 PE = 3 SV = 1
comp44384_c1	LOB domain-containing protein 41 OS = Arabidopsis thaliana GN = LBD41 PE = 2 SV = 1
comp43977_c0	--
comp43311_c0	predicted protein [Arabidopsis lyrata subsp. lyrata] > gi|297331837|gb|EFH62256.1| predicted protein [Arabidopsis lyrata subsp. lyrata]
comp47017_c0	uncharacterized protein LOC100814873 [Glycine max] > gi|255637360|gb|ACU19009.1| unknown [Glycine max]

The DEGs from transcriptome and DGE sequencing were subjected to a search against GO and KEGG databases. The results showed that many of the DEGs were involved in metabolic processes related to chlorophyll, plant hormone metabolism, carbohydrate metabolism, signal transduction and cell wall construction. The results were consistent with Qi’s (2013) [12], and suggest that calyx persistence in Korla fragrant pear is regulated by many genes.

## Conclusion

More than 50 DEGs were obtained through transcriptome and DGE sequencing. These DEGS were related to cell wall metabolism, plant hormone metabolism, stress response, zinc finger protein, and lipid-transfer protein. Analysis of the functions and metabolic pathways of the DEGs indicated that calyx abscission in Korla fragrant pear was a metabolic process induced by a variety of genes related to cell wall metabolism and regulated by multiple plant hormones. Our laboratory is currently researching the protein function of the DEGs in Korla fragrant pear.

## Methods

### Plant material

**Fig. 8:**
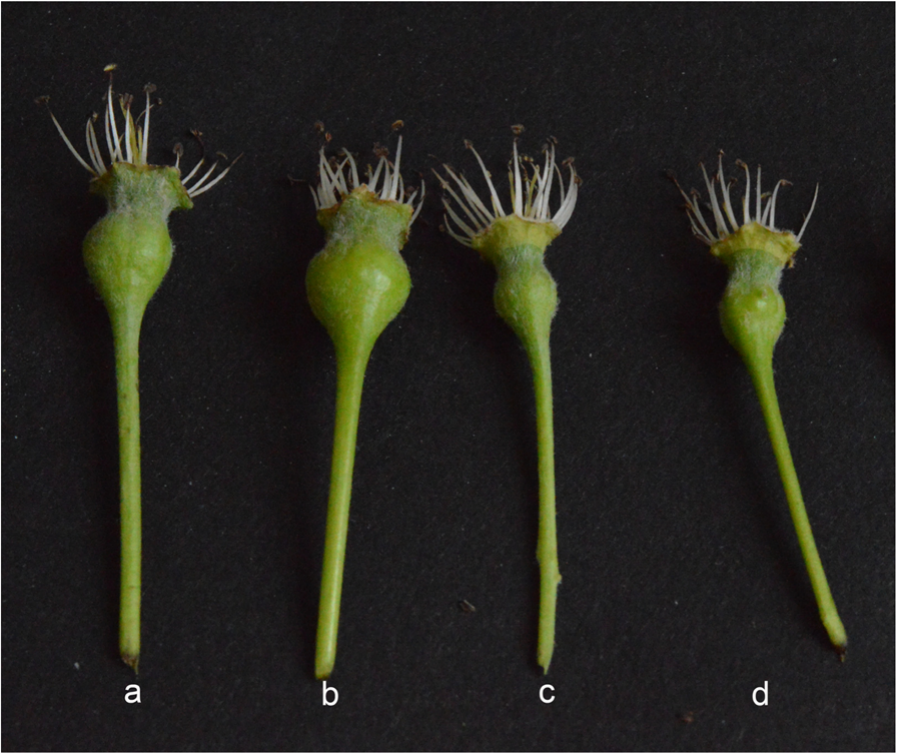
Flowers with persistent and deciduous calyx of Korla fragrant pear. The **a** and **b** indicate flowers with persistent calyx. The **c** and **d** indicate flowers with deciduous calyx

Three trees with high vigor and three trees with low vigor were selected in spring 2013 at the Shayidong Horticulture Field, Korla, Xinjiang Province. Flowers were collected from each tree at the early bloom, full bloom, and late bloom stages. The first flower to open in clusters on trees with high vigor has a persistent calyx (Fig. 8a, b). The fourth flower to open in clusters from trees with low vigor has a deciduous calyx (Fig. 8c, d). The flowers were immediately frozen in liquid N and stored at −80 °C.

### Transcriptome sequencing

Solexa/Illumina sequencing was carried out by Novogene, Beijing, China. Total RNA was extracted from the flower samples using RNAout 1.0 (Tianenze, Beijing, China). The RNA degradation and contamination was monitored on 1 % agarose gels. The purity of the RNA was checked with a NanoPhotometer® (IMPLEN, CA, USA). The RNA concentration was measured using a Qubit®RNA Assay Kit and a Qubit®2.0 Fluorometer (Life Technologies, CA, USA). The RNA integrity was assessed using an RNA Nano 6000 Assay Kit and an Agilent Bioanalyzer 2100 system (Agilent Technologies, CA, USA). After quality inspection, the RNA from flowers at the early, full, and late bloom stages were combined by calyx type. The combined RNA sample from flowers with a persistent calyx will be referred to as SC_hua. The combined RNA sample from flowers with a deciduous calyx will be referred to as TL_hua. These RNA samples were used for transcriptome sequencing. Three biological replicates were used.

The RNA preparations used 3 μg RNA per sample. Sequencing libraries were generated using NEBNext®Ultra™ RNA Library Prep Kit for Illumina® (NEB, USA) following the manufacturer’s recommendations. Index codes were added to attribute sequences in each sample. Briefly, mRNA was purified from total RNA using poly-T oligo-attached magnetic beads. Fragmentation was carried out using divalent cations under elevated temperature in NEBNext First Strand Synthesis Reaction Buffer (5x). First strand cDNA was synthesized using random hexamer primer and M-MuLV Reverse Transcriptase (RNase H^−^). Second strand cDNA synthesis was subsequently performed using DNA Polymerase I and RNase H. Remaining overhangs were converted into blunt ends via exonuclease/polymerase activities. After adenylation of the 3’ ends, the DNA fragments were ligated with the NEBNext Adaptor with hairpin loop structure. The library fragments were purified with AMPure XP (Beckman Coulter, Beverly, USA) in order to select cDNA fragments with lengths of 150 ~ 200 bp. The size-selected, adaptor-ligated cDNA was mixed with 3 μl USER Enzyme (NEB, USA) at 37 °C for 15 min followed by 5 min at 95 °C before PCR. The PCR was performed with Phusion High-Fidelity DNA polymerase, universal PCR primers and Index (X) Primer. The PCR products were purified (AMPure XP system) and the library quality was assessed using an Agilent Bioanalyzer 2100.

The clustering of the index-coded samples was performed on a cBot Cluster Generation System using TruSeq PE Cluster Kit v3-cBot-HS (Illumia) according to the manufacturer’s instructions. After cluster generation, the library preparations were sequenced on an Illumina Hiseq 2000 platform and paired-end reads were generated.

### Data analysis of transcriptome sequencing

Raw data (raw reads) in fastq format were first processed through in-house Perl scripts. Clean data (clean reads) were obtained by removing reads containing adapter sequences, reads containing poly-N, and low quality reads. The Q20, Q30, GC-content, and sequence duplication level of the clean data were calculated. All downstream analyses were based on clean data with high quality.

The left files (read1 files) from all libraries/samples were pooled into one large left.fq file. The right files (read2 files) were pooled into one large right.fq file. Transcriptome assembly was accomplished based on the left.fq and right.fq files using Trinity [63]. The min_kmer_cov was set at 2 and all other parameters were set at default. Gene function was annotated based on the following databases: NR (NCBI non-redundant protein sequences); NT (NCBI non-redundant nucleotide sequences); PFAM (Protein family); KOG/COG (Clusters of Orthologous Groups of proteins); SwissProt (A manually annotated and reviewed protein sequence database); KO (KEGG Ortholog database); GO (Gene Ontology).

### DGE sequencing

The RNA was extracted from sepals and ovaries at the early, full, and late bloom stages. The RNA was combined by calyx type. The combined RNA sample from sepals with a persistent calyx will be referred to as SC_ep. The combined RNA sample from sepals with a deciduous calyx will be referred to as TL _ep. The combined RNA sample from ovaries with a persistent calyx will be referred to as SC_zf. The combined RNA sample from ovaries with a deciduous calyx will be referred to as TL _zf. The methods of RNA extraction, RNA quantification, RNA qualification, clustering, and sequencing were the same as those described above for transcriptome sequencing.

### Differential expression analysis

#### Samples with biological replicates

Differential expression analysis of two conditions/groups was performed using the DESeqR package (1.10.1). The DESeq provides statistical routines for determining differential expression in digital gene expression data using a model based on negative binomial distribution. The resulting P values were adjusted using Benjamini and Hochberg’s approach for controlling the false discovery rate. Genes were considered to be differentially expressed if DESeq found the adjusted P-value to be <0.05.

#### Samples without biological replicates

Prior to differential gene expression analysis, the read counts for each sequenced library were adjusted using edgeR software through one scaling normalized factor. Differential expression analysis of two samples was performed using DEGseq R package (2010). The P value was adjusted using the q value [64]. The q value < 0.005&|log2 (fold change)| > 1 was set as the threshold for significantly differential expression.

### GO enrichment analysis

Gene Ontology (GO) enrichment analysis of the differentially expressed genes (DEGs) was implemented by GOseq R packages based on Wallenius non-central hyper-geometric distribution [65] which can be adjusted for gene length bias in DEGs.

### KEGG pathway enrichment analysis

KEGG [66] is a database resource for understanding high-level functions and utilities of biological systems (e.g., cell, organism, and ecosystem), from molecular-level information, especially large-scale molecular datasets generated by genome sequencing and other high-throughput experimental technologies (http://www.genome.jp/kegg/). We used KOBAS [67] software to test the statistical enrichment of differentially expressed genes in KEGG pathways.

### Protein Protein Interaction (PPI)

The sequences of the DEGs were BLASTx against the genome of a related species (the PPI of which exists in the STRING database: http://string-db.org/) to get the predicted PPIs of these DEGs. The PPIs were visualized in Cytoscape [68].

### Real-time quantitative PCR

The expression of five genes (Gene ID: comp36863_c0, comp41728_c0, comp46544_c0, comp49798_c0, and comp49614_c0) that might be associated with calyx persistence in Korla Fragrant Pear were analyzed by qRT-PCR. Total RNA was separately extracted from the full flowers, sepals and ovaries using RNAout 1.0 (Tianenze, Beijing, China) at the early bloom, full bloom, and late bloom stages. The RNA samples were from (i) sepals with persistent calyx, (ii) ovaries with persistent calyx, (iii) sepals with deciduous calyx, (iv) ovaries with deciduous calyx, (v) full flowers with deciduous calyx, and (vi) full flowers with persistent calyx. Each group had three biological replications. Gene-specific primers were designed according to the reference unigene sequences using Primer Premier 5.0 (Table 12). The synthesis of cDNA was performed using a Reverse Transcriptase M-MLV kit (TaKaRa, Dalian, China). Real-time quantification was performed using a CFX manager (Bio-Rad, USA) and the SYBR Green Real-time PCR Master Mix (Toyobo, Osaka, Japan). The protocol of real-time PCR was as follows: initiation with a 30 s pre-denaturation at 95 °C followed by 40 cycles of amplification with 5 s of denaturation at 95 °C, 10 s of annealing at 56 °C, 15 s of extension at 72 °C and reading the plate for fluorescence data collection at 65 °C. A melting curve was performed from 65 to 95 °C to check the specificity to the amplified product. Each reaction was repeated three times. Korla fragrant pear actin gene (forward: 5’-CCATCCAGGCTGTTCTCTC-3’, and reverse: 5’-GCAAGGTCCAGACGAAGG -3’) was used as a normalizer.

**Table 12:** Primer for qRT-PCR

Primer ID	Primer sequences (5’ to 3’)
comp36863_c0	AACTACTTCTCGCCATCGT
	TGTTCTTGCTCTTCCTCGT
comp41728_c0	GCGTGGAGGTAGGAGAAC
	CAAGAAGGGCAGCATAGA
comp46544_c0	GAGGAGGAAATGAAGAGG
	ATCAATCAAACAGGTGGC
comp49798_c0	AATAAACTGCCCAAACGA
	ACAAGCAACCCAATCTCA
comp49614_c0	CACGAAGTGGTCGGAAAG
	GGAGAATGCGTCACAGTAG

### Availability of supporting data

Illumina sequencing data from ‘Korla Fragrant Pear’ SC_hua, TL_hua, SC_zf, TL_zf, SC_ep, and TL_ep were deposited in the NCBI SRA database under accession number SRP066513, bioProject accession: PRJNA303067 (http://www.ncbi.nlm.nih.gov/bioproject/303067). The release time is 2016-11-21 00:00:00. All the supporting data have been provided as Additional files (1, 2, 3, 4, 5, 6 and 7).

## Additional files

Additional file 1:
**GO enrichment of DEGs in SC_hua vs TL_hua.** GO_accession: The ID of nodal point. Description: GO description. Term_type: The category of GO. Over_represented_pValue: The pValue of enrichment. Corrected_pValue: The pValue is after correction. DEG_item: The DEGs related to this GO. DEG_list: All the DEGs annotated in GO. Bg_item: The background genes related to this GO. Bg_list: The background genes annoted in this GO. Gene_names: The DEGs’ ID related to this GO. (XLS 130 kb) Additional file 2:
**GO enrichment of up-regulated DEGs in SC_hua vs TL_hua.** GO_accession: The ID of nodal point. Description: GO description. Term_type: The category of GO. Over_represented_pValue: The pValue of enrichment. Corrected_pValue: The pValue is after correction. DEG_item: The DEGs related to this GO. DEG_list: All the DEGs annotated in GO. Bg_item: The background genes related to this GO. Bg_list: The background genes annoted in this GO. Gene_names: The DEGs’ ID related to this GO. (XLS 82 kb) Additional file 3:
**The top 20 KEGG pathways enrichment of DEGs in SC_hua vs TL_hua.** (XLS 1 kb) Additional file 4:
**The top 20 KEGG pathways enrichment of up DEGs in SC_hua vs TL_hua.** (XLS 512 bytes) Additional file 5:
**The top 20 KEGG pathways enrichment of down DEGs in SC_hua vs TL_hua.** (XLS 977 bytes) Additional file 6:
**The top 20 KEGG pathways enrichment of DEGs in SC_ep vs TL_ep.** (XLS 1 kb) Additional file 7:
**The top 20 KEGG pathways enrichment of DEGs in SC_zf vs TL_zf.** (XLS 1 kb)

## Abbreviations

SC_hua: flowers with persistent calyx
SC_ep: sepals of flowers with persistent calyx
SC_ zf: ovaries of flowers with persistent calyx
TL_hua: flowers of flowers with deciduous calyx
TL_ep: sepals of flowers with deciduous calyx
TL_zf: ovaries with deciduous calyx
DEGs: differentially expressed genes
qRT-PCR: real-time quantitative PCR
DGE: digital gene expression
RNA-Seq: high-throughput sequencing of RNA
NR: NCBI non-redundant protein sequences
NT: NCBI nucleotide sequences
KEGG: Kyoto encyclopedia of genes and genomes
SwissProt: a manually annotated and reviewed protein sequence database
PFAM: protein family
GO: gene ontology
KOG/COG: clusters of orthologous groups of proteins
CC: the cellular component category
BP: the biological process category
MF: the molecular function category
